# Supplementary material for: Unsupervised data-driven stratification of mentalizing heterogeneity in autism
Source: Sci Rep. 2016 Oct 18;6:35333. doi: 10.1038/srep35333 (PMC5067562; doi:10.1038/srep35333)
Supplement: Supplementary Information [file srep35333-s1.pdf]

Supplementary Figures and Table Legend for

Title: Unsupervised data-driven stratification of mentalizing heterogeneity in autism

Authors: Michael V. Lombardo[1,2]*, Meng-Chuan Lai[2,3,4]*, Bonnie Auyeung[2,5], Rosemary J. Holt[2], Carrie Allison[2], Paula Smith[2], Bhismadev Chakrabarti[2,6], Amber N. V. Ruigrok[2], John Suckling[7,8], Edward T. Bullmore[7,8], MRC AIMS Consortium[2,7,9,10,11]**, Christine Ecker[9,12], Michael C. Craig[9,13], Declan G. M. Murphy[9], Francesca Happé[11], & Simon Baron-Cohen[2,8]

Affiliations:
[1] Center for Applied Neuroscience, Department of Psychology, University of Cyprus, Nicosia, Cyprus
[2] Autism Research Centre, Department of Psychiatry, University of Cambridge, Cambridge, UK
[3] Child and Youth Mental Health Collaborative at the Centre for Addiction and Mental Health and The Hospital for Sick Children, Department of Psychiatry, University of Toronto, Toronto, Canada
[4] Department of Psychiatry, National Taiwan University Hospital and College of Medicine, Taipei, Taiwan
[5] School of Philosophy, Psychology and Language Sciences, Department of Psychology, University of Edinburgh, Edinburgh, UK
[6] Centre for Integrative Neuroscience and Neurodynamics, School of Psychology and Clinical Language Sciences, University of Reading, Reading, UK
[7] Brain Mapping Unit, Department of Psychiatry, University of Cambridge, Cambridge, UK
[8] Cambridgeshire and Peterborough NHS Foundation Trust, Cambridge, United Kingdom
[9] Sackler Institute for Translational Neurodevelopment, Department of Forensic and Neurodevelopmental Sciences, Institute of Psychiatry, Psychology and Neuroscience, King's College London, London, UK
[10] Autism Research Group, Department of Psychiatry, University of Oxford
[11] MRC Social, Genetic, and Developmental Psychiatry Centre, Institute of Psychiatry, Psychology & Neuroscience, King's College London, London, UK
[12] Department of Child & Adolescent Psychiatry, Psychosomatics and Psychotherapy, University Hospital Frankfurt am Main, Goethe-University, Frankfurt am Main, Germany
[13] National Autism Unit, Bethlem Royal Hospital, SLAM NHS Foundation Trust, UK

Corresponding Author: Michael V. Lombardo (mvlombardo@gmail.com), University of Cyprus, Department of Psychology, 1 Panepistimiou Avenue, Aglantzia, Nicosia 1678, Cyprus

Running Title: Mentalizing subgroups in autism

* Equal contributions

** The Medical Research Council Autism Imaging Multicentre Study Consortium (MRC AIMS Consortium) is a UK collaboration between the Institute of Psychiatry (IoP) at King's College, London, the Autism Research Centre, University of Cambridge, and the Autism Research Group, University of Oxford. The Consortium members are in alphabetical order: Anthony J. Bailey (Oxford), Simon Baron-Cohen (Cambridge), Patrick F. Bolton (IoP), Edward T. Bullmore (Cambridge), Sarah Carrington (Oxford), Marco Catani (IoP), Bhismadev Chakrabarti (Cambridge), Michael C. Craig (IoP), Eileen M. Daly (IoP), Sean C. L. Deoni (IoP), Christine Ecker (IoP), Francesca Happé (IoP), Julian Henty (Cambridge), Peter Jezzard (Oxford), Patrick Johnston (IoP), Derek K. Jones (IoP), Meng-Chuan Lai (Cambridge), Michael V. Lombardo (Cambridge), Anya Madden (IoP), Diane Mullins (IoP), Clodagh M. Murphy (IoP), Declan G. M. Murphy (IoP), Greg Pasco (Cambridge), Amber N. V. Ruigrok (Cambridge), Susan A. Sadek (Cambridge), Debbie Spain (IoP), Rose Stewart (Oxford), John Suckling (Cambridge), Sally J. Wheelwright (Cambridge), Steven C. Williams (IoP), and C. Ellie Wilson (IoP).

Supplementary Figure 1: Item-difficulty Patterning Across TD Subgroups.
Panels A (TD Discovery) and B (TD Replication) show item-difficulty profiles (i.e., percentage of subjects within a subgroup that answer the item correctly) for each ASC subgroup denoted by the different colored lines. Panels C and D show correlation matrices from item-difficulty between subgroups. Asterisks indicate specific comparisons that pass FDR q<0.05 correction for multiple comparisons.

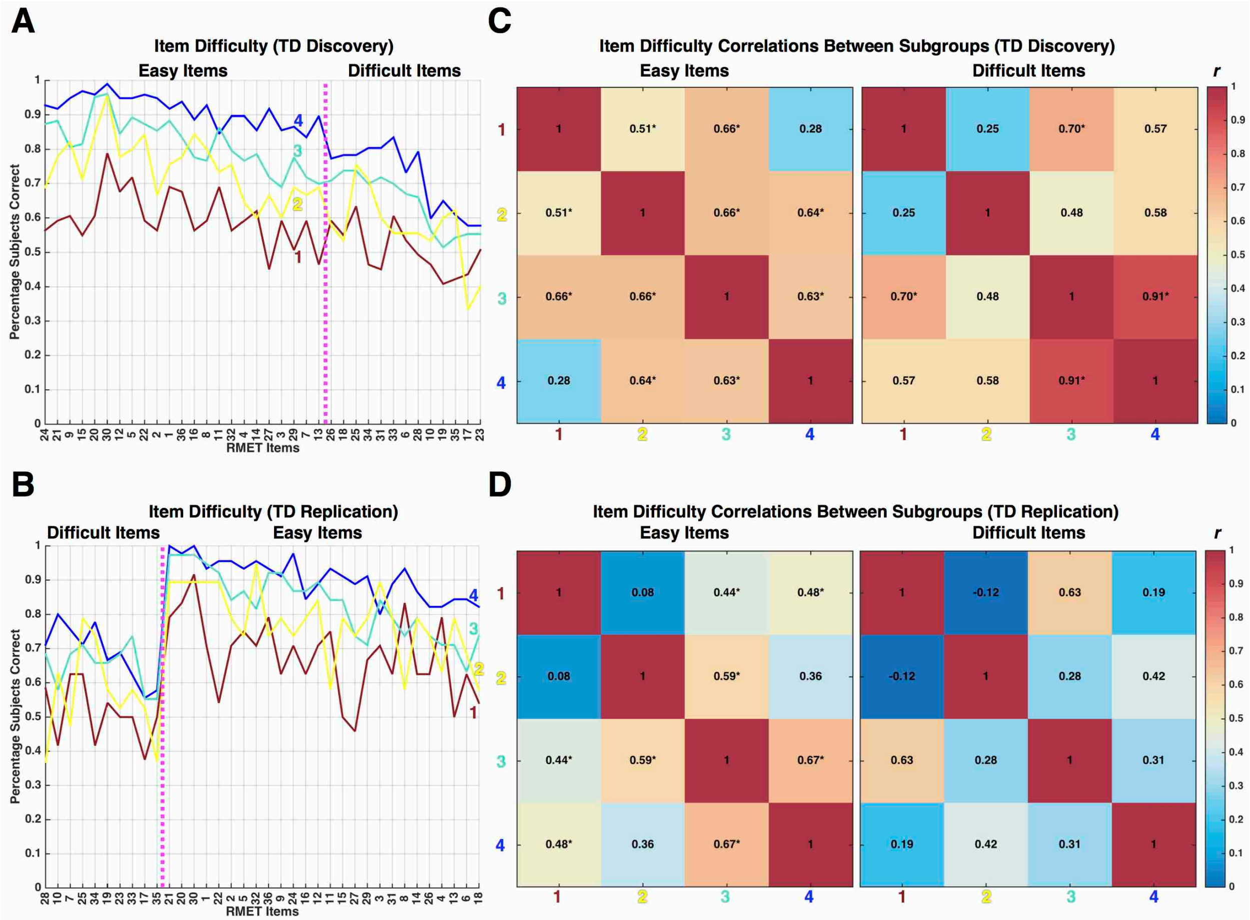

Supplementary Figure 2: TD between-subject dissimilarity of RMET response patterns. This figure depicts between-subject dissimilarity matrices in TD for the easy item (A) or difficult item (B) subsets. Cooler colors indicate more between-subject similarity, whereas hotter colors indicate more between-subject dissimilarity. Each cell of the matrices represents the dissimilarity between a pair of subjects. The rows and columns are arranged by subgroup rank order and Discovery and Replication datasets are adjacent to each other and denoted above the rows and columns by D and R. The black outlines delineate between-subject dissimilarities within a particular subgroup.

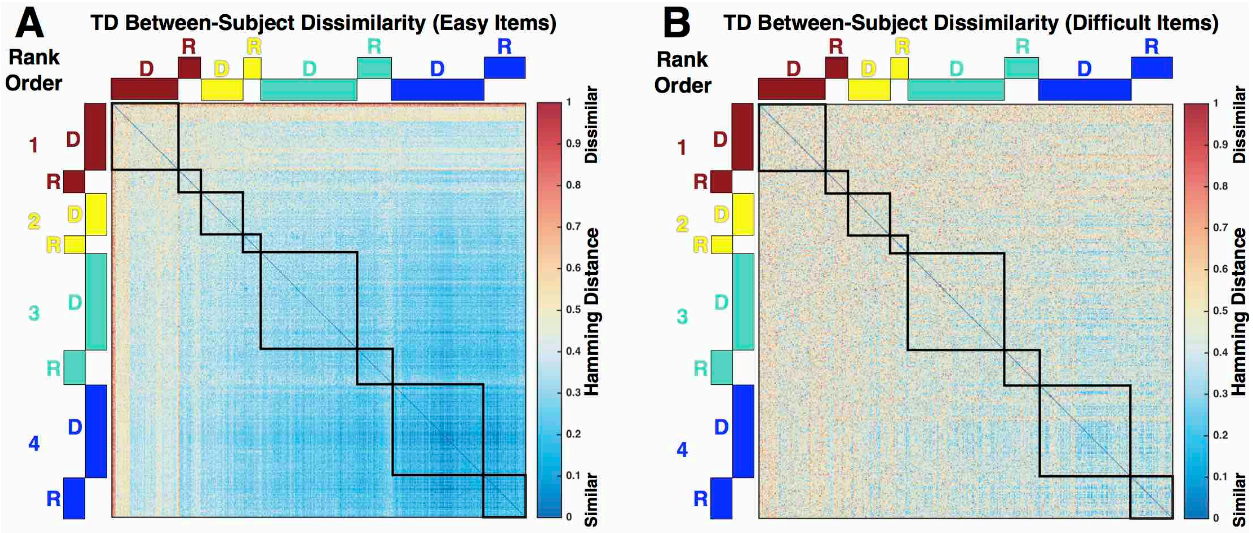

Supplementary Figure 3: Confusion matrices for multi-class classifier predictions of TD subgroup membership.

Confusion matrices show counts of actual TD subgroup membership along the rows and classifier predicted subgroup membership along the columns. The coloring of cells in the confusion matrices represents the percentage of actual subgroup individuals predicted within each subgroup category. Above the matrices are descriptions of which dataset was used for training and testing.

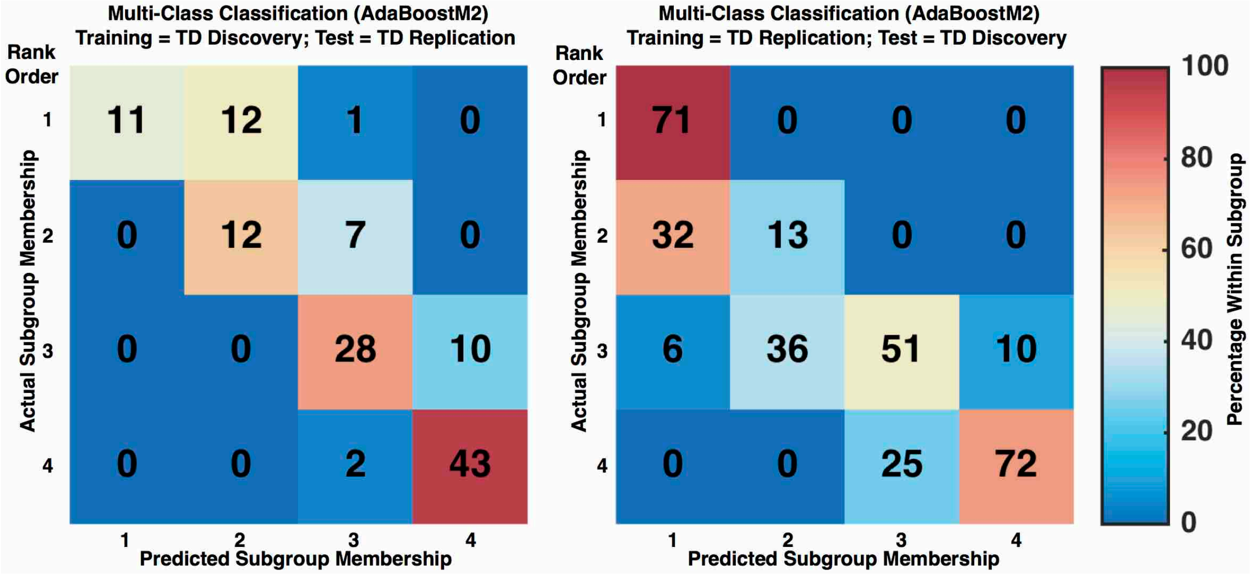

Supplementary Figure 4: Multi-class classifier performance

This figure shows the histogram of the null distribution of classifier accuracy when labels were randomly shuffled (10,000 iterations). The true accuracy level under the real labels is shown as a red line. Panel A shows performance for the analysis on ASC subgroups, whereas panel B shows performance for TD subgroups.

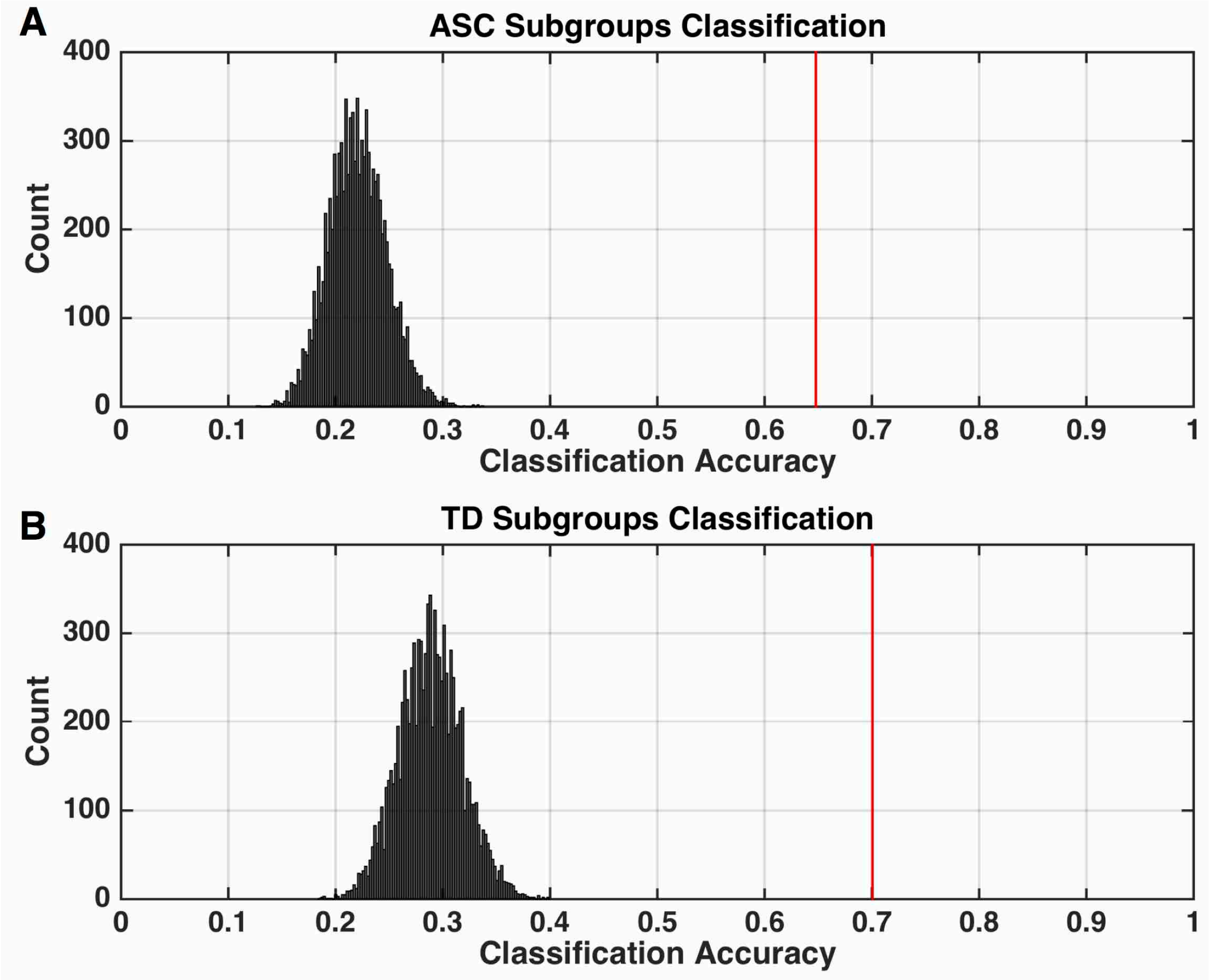

Supplementary Figure 5: Verbal IQ

This figure shows verbal IQ data as a boxplot with dots overlaid to represent individual subject's data points. It also shows heatmaps of effect size for comparisons made within ASC subgroups, within TD subgroups, and comparisons between ASC and TD subgroups. An asterisk next to effect sizes indicates that this comparison passed Bonferroni correction for multiple comparisons.

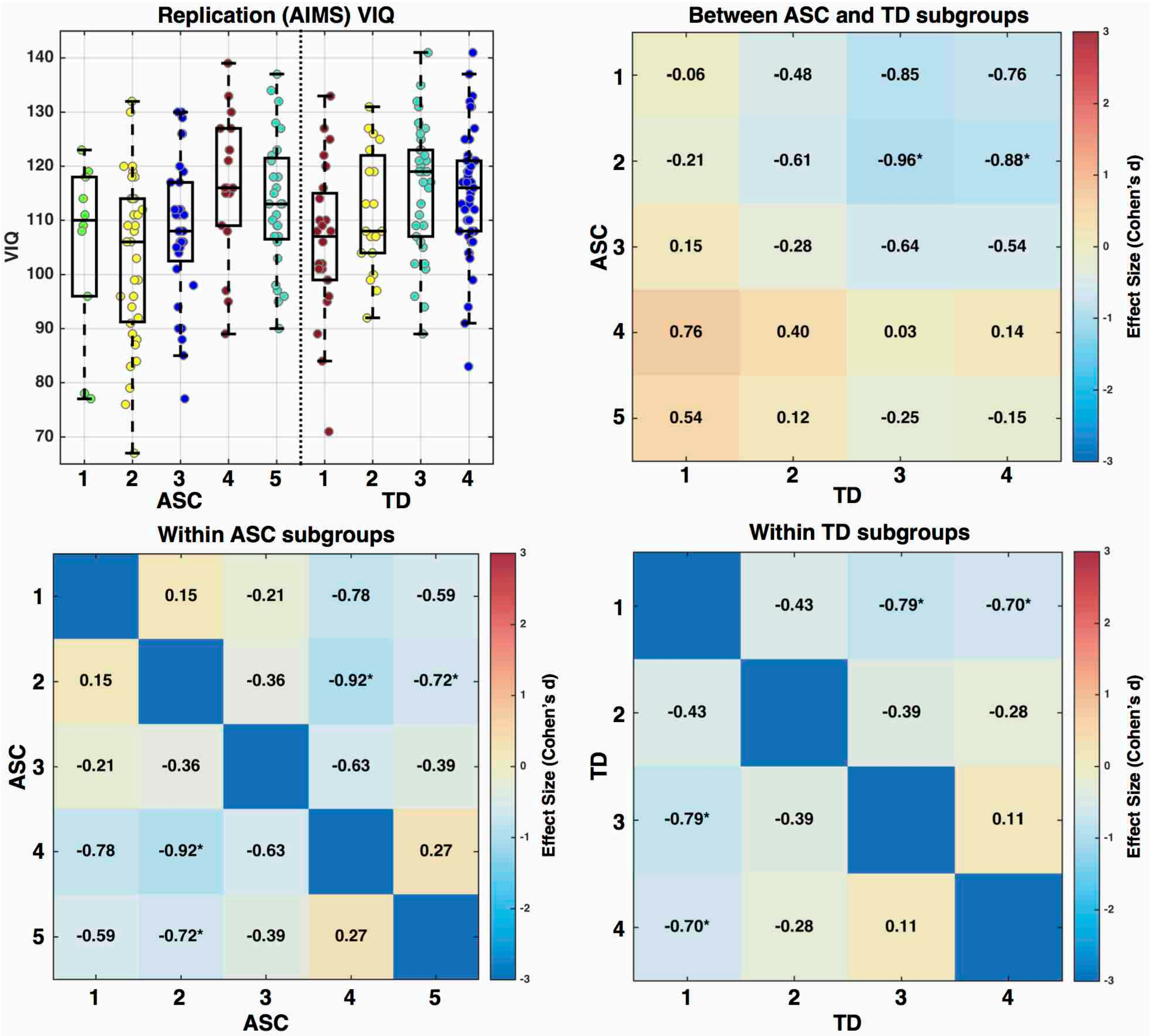

Supplementary Figure 6: Age

This figure shows age data as a boxplot with dots overlaid to represent individual subject's data points. It also shows heatmaps of effect size for comparisons made within ASC subgroups, within TD subgroups, and comparisons between ASC and TD subgroups. Panel A shows data from the Discovery (CARD) dataset, whereas panel B shows data from the Replication (AIMS) dataset. An asterisk next to effect sizes indicates that this comparison passed Bonferroni correction for multiple comparisons.

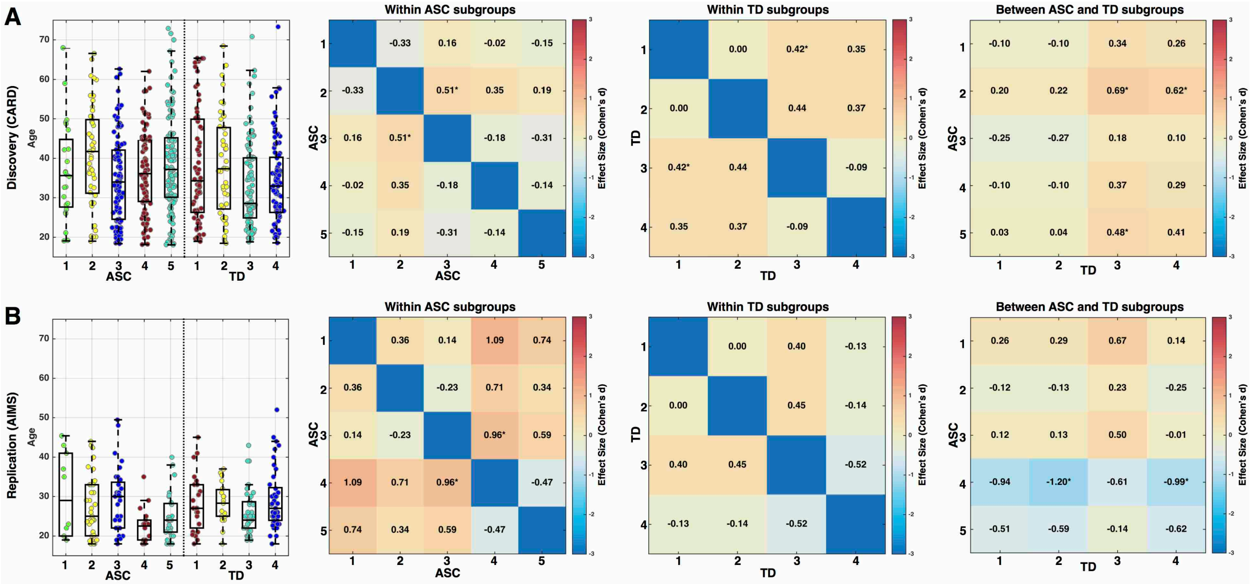

Supplementary Figure 7: AQ

This figure shows AQ data as a boxplot with dots overlaid to represent individual subject's data points. It also shows heatmaps of effect size for comparisons made within ASC subgroups, within TD subgroups, and comparisons between ASC and TD subgroups. Panel A shows data from the Discovery (CARD) dataset, whereas panel B shows data from the Replication (AIMS) dataset. An asterisk next to effect sizes indicates that this comparison passed Bonferroni correction for multiple comparisons.

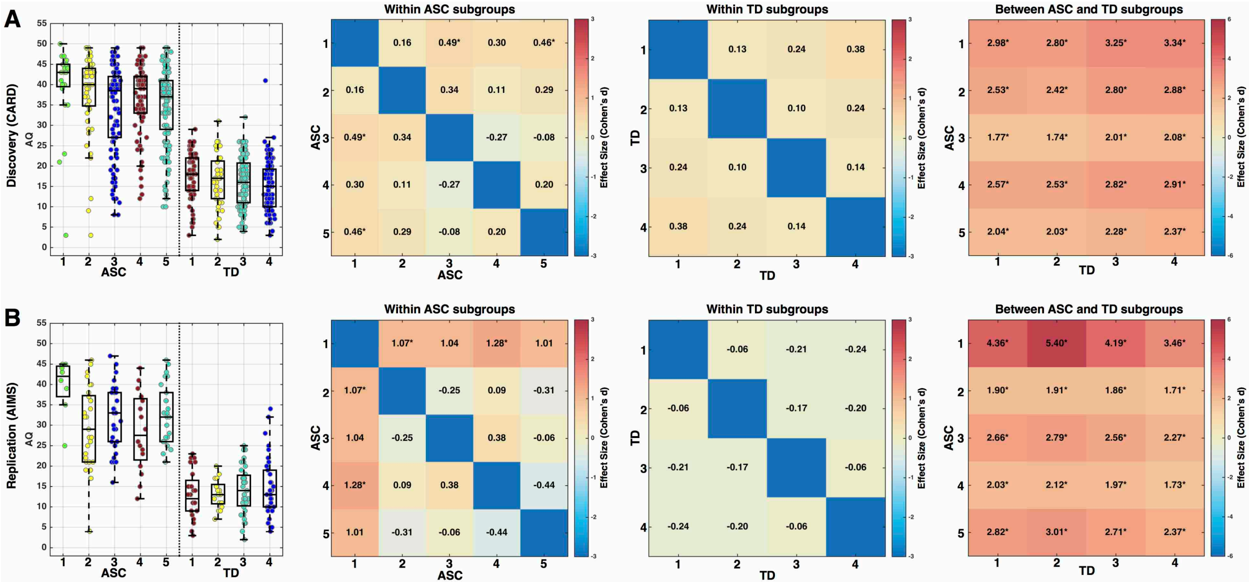

Supplementary Figure 8: EQ

This figure shows EQ data as a boxplot with dots overlaid to represent individual subject's data points. It also shows heatmaps of effect size for comparisons made within ASC subgroups, within TD subgroups, and comparisons between ASC and TD subgroups. Panel A shows data from the Discovery (CARD) dataset, whereas panel B shows data from the Replication (AIMS) dataset. An asterisk next to effect sizes indicates that this comparison passed Bonferroni correction for multiple comparisons.

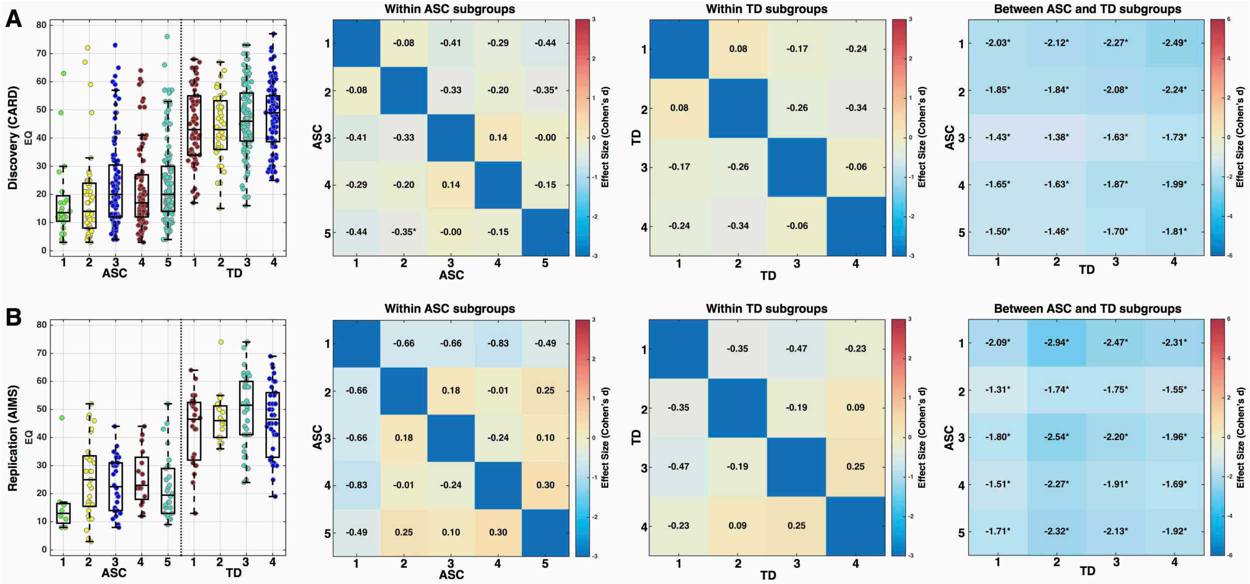

Supplementary Figure 9: BDI and BAI

This figure shows BDI (panel A) and BAI (panel B) data as boxplots with dots overlaid to represent individual subject's data points. It also shows heatmaps of effect size for comparisons made within ASC subgroups, within TD subgroups, and comparisons between ASC and TD subgroups. An asterisk next to effect sizes indicates that this comparison passed Bonferroni correction for multiple comparisons.

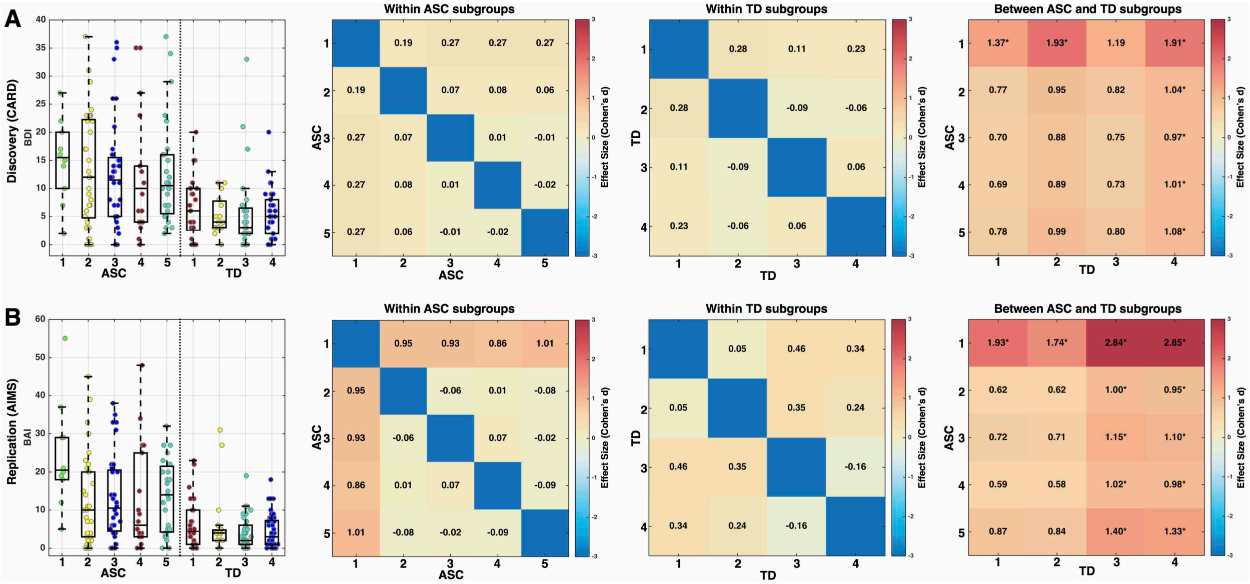

Supplementary Table

The supplementary tables show detailed statistical information for comparisons across all variables examined in the manuscript.
